# Supplementary material for: Modulation of neural gene networks by estradiol in old rhesus macaque females
Source: GeroScience. 2024 Mar 20;46(6):5819–41. doi: 10.1007/s11357-024-01133-z (PMC11493911; doi:10.1007/s11357-024-01133-z)
Supplement: Supplementary file 1 — Supplementary file1 (DOCX 7700 KB) [file 11357_2024_1133_MOESM1_ESM.docx]

**Supplemental material**

**Table S1 – Significant Results.** The number of significant results from each of the various analyses that were computed. Numbers in the parentheses represent the number of results with recognizable gene symbols that were used in network analysis.

| **Analysis** | **Tissue** | | |
| --- | --- | --- | --- |
|  | **Occipital Cortex** | **Prefrontal Cortex** | **Both** |
| Exon Usage (FDR *p* < 0.05) | 20 (15) | 0 | 0 |
| Expression (FDR *p* < 0.05) | 150 (131) | 128 (110) | 1 (1) |
| DNA methylation (Sidak’s *p* < 0.05) | 254 (207) | 457 (400) | 18 (18) |
| Expression & DNA methylation | 0 | 5 (5) | 5 (5) |

FIGURE S1

**A B**


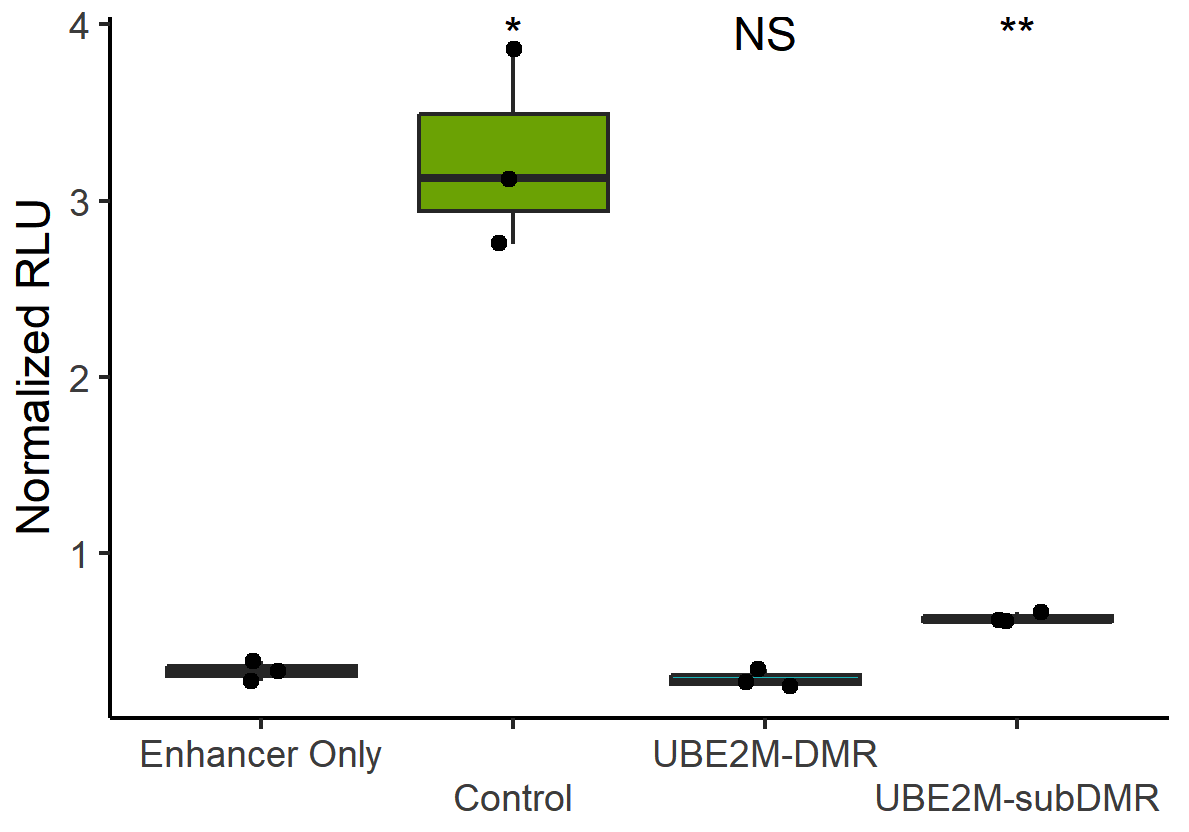
**
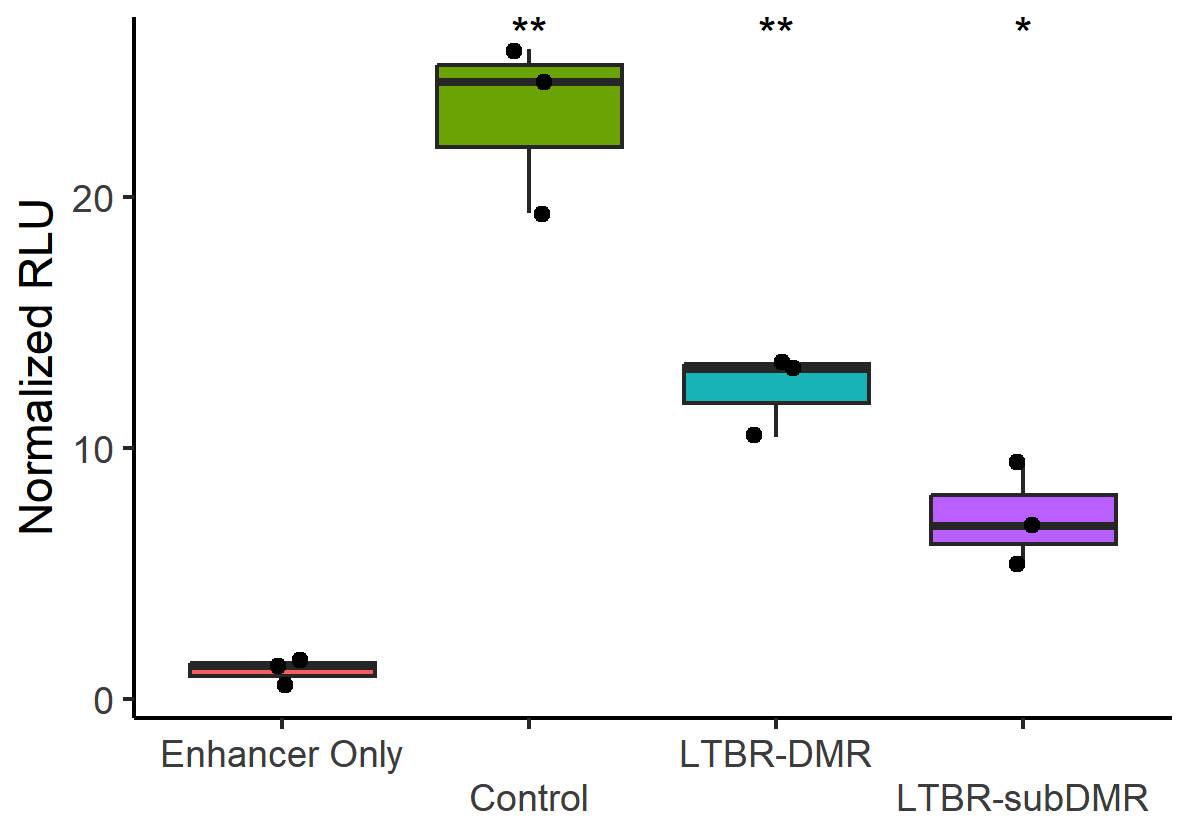
**

**Fig.S1 – Promoter assay** (p-values are based on a Student’s *t*-test with “Enhancer Only” group as the reference plasmid with only an enhancer element, and Control as the positive control plasmid containing a promoter and an enhancer, where *** = *p* < 0.001, ** = *p* < 0.01, * = *p* < 0.05, and NS = non-significant)

FIGURE S2.


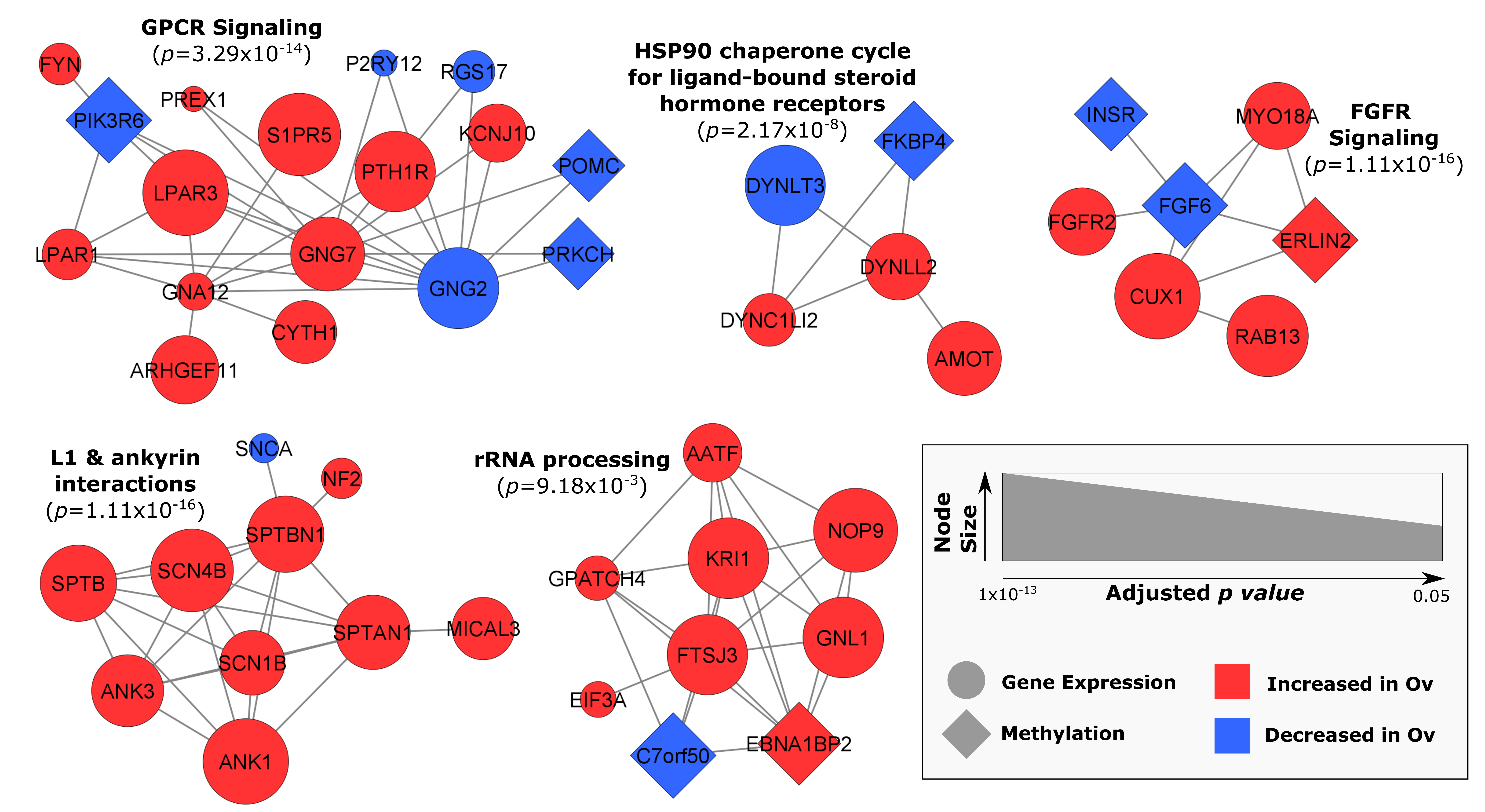


**Fig. S2 – Biological networks of ovariectomy-related changes in gene expression and methylation in the rhesus macaque prefrontal cortex (PFC)**. Protein interactions were obtained from STRING’s protein interaction database. MCODE was used to find tightly connected clusters of interactions that are labeled according to function defined in Gene Ontology biological processes (enrichment p-value listed). The color of the nodes reflect the direction of effect while the shape of the nodes reflect which omics analysis the gene was identified in. The size of the node reflects statistical significance with larger nodes being more significant

FIGURE S3


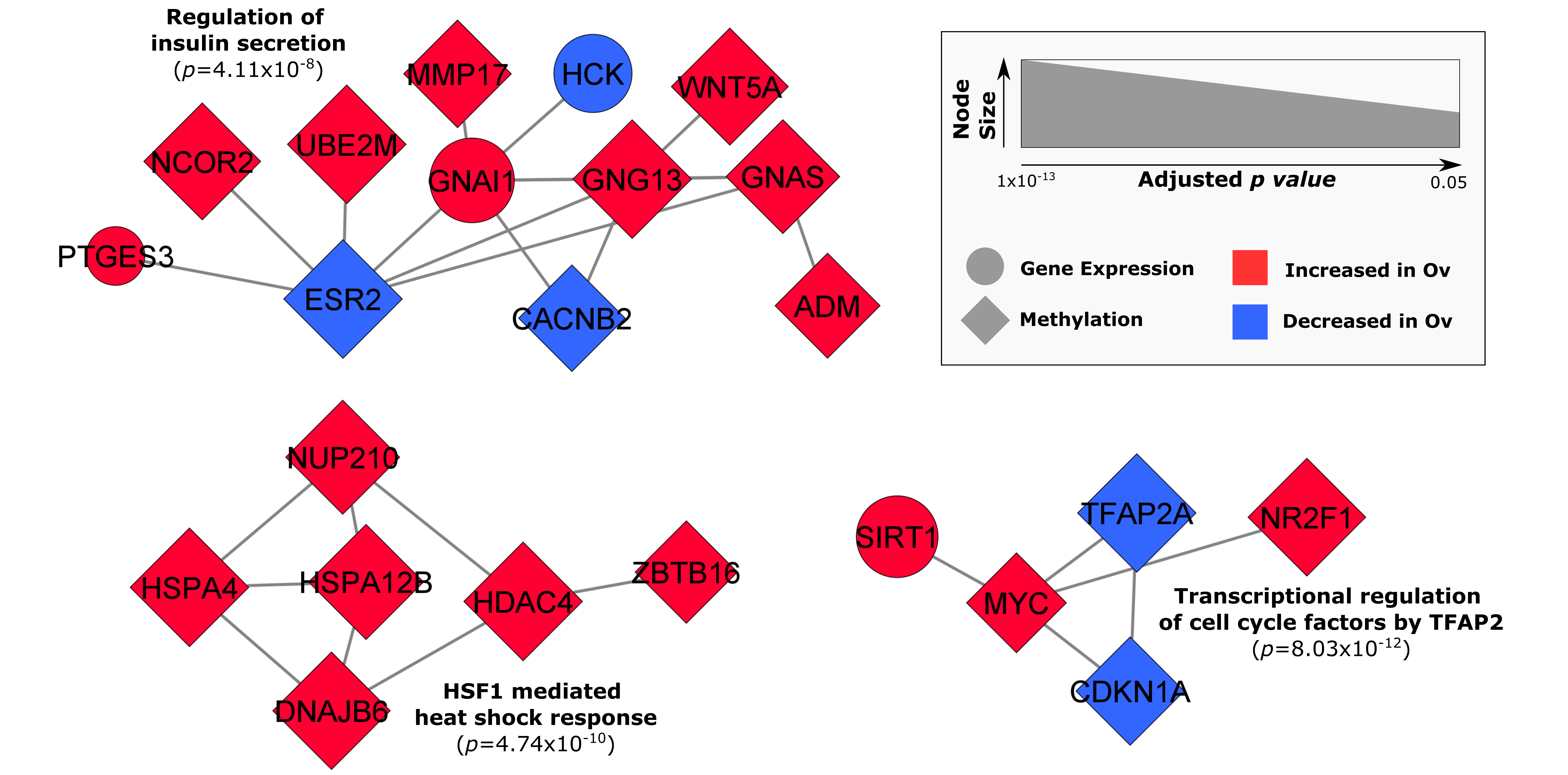


**Fig. S3 – Biological networks of ovariectomy-related changes in gene expression and methylation in rhesus macaque Occipital cortex (OC)**. Protein interactions were obtained from STRING’s protein interaction database. MCODE was used to find tightly connected clusters of interactions that are labeled according to function defined in Gene Ontology biological processes (enrichment p-value listed). The color of the nodes reflect the direction of effect while the shape of the nodes reflect which omics analysis the gene was identified in. The size of the node reflects statistical significance with larger nodes being more significant

FIGURE S4


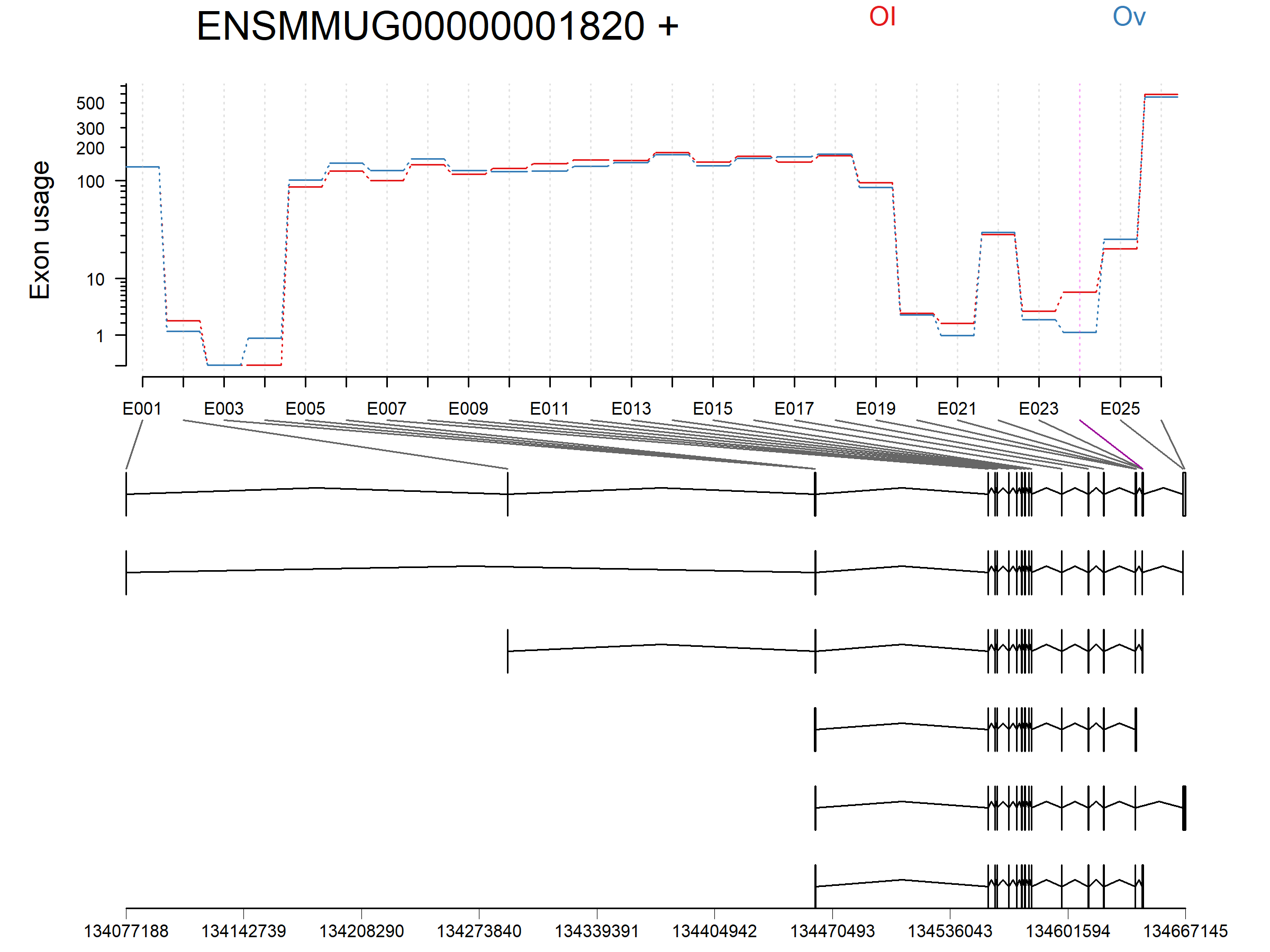


**Fig. S4 – Differential Exon Usage for RGS6.** Exon E024 is downregulated with Ov, meaning the isoform highlighted in red is expressed in higher proportions with Ov
